# Optimizing Systems for Robust Heterologous Production of Biosurfactants Rhamnolipid and Lyso-Ornithine Lipid in *Pseudomonas putida* KT2440

**DOI:** 10.3390/molecules29143288

**Published:** 2024-07-11

**Authors:** Xuelian Li, Zhili Yang, Jianhua Liu

**Affiliations:** Systems Biology, School for Marine Science and Technology, Zhejiang Ocean University, Zhoushan 316022, China; lixuelian@zjou.edu.cn (X.L.); yangzhili@zjou.edu.cn (Z.Y.)

**Keywords:** biosurfactant, heterologous expression, lyso-ornithine lipid, *Pseudomonas putida*, rhamnolipid

## Abstract

Surfactants are amphiphilic molecules that are capable of mixing water and oil. Biosurfactants are eco-friendly, low-toxicity, and stable to a variety of environmental factors. Optimizing conditions for microorganisms to produce biosurfactants can lead to improved production suitable for scaling up. In this study, we compared heterologous expression levels of the luminescence system *luxCDABE* operon controlled by regulatable promoters araC-P_BAD_ and its strong version araC-P_BAD-SD_ in *Escherichia coli* K12, *Pseudomonas aeruginosa* PAO1, and *P. putida* KT2440. Real-time monitoring of luminescence levels in the three strains indicated that *luxCDABE* controlled by araC-P_BAD-SD_ promoter with 0.2% arabinose supplementation in *P. putida* produced the highest level of luminescence. By using the araC-P_BAD-SD_ promoter-controlled *rhlAB* expression in *P. putida*, we were able to produce mono-rhamnolipid at a level of 1.5 g L^−1^ when 0.02% arabinose was supplemented. With the same system to express *olsB*, lyso-ornithine lipid was produced at a level of 10 mg L^−1^ when 0.2% arabinose was supplemented. To our knowledge, this is the first report about optimizing conditions for lyso-ornithine lipid production at a level up to 10 mg L^−1^. Taken together, our results demonstrate that regulatable araC-P_BAD-SD_ promoter in *P. putida* KT2440 is a useful system for heterologous production of biosurfactants.

## 1. Introduction

Biosurfactants are surface-active compounds containing both hydrophobic and hydrophilic domains that are capable of decreasing surface tension and interfacial tension. Biosurfactants have different chemical structures or families [1], such as glycolipids including rhamnolipids, sophorolipids, and mannosylerythritol lipids [2]; lipopeptides including surfactin, iturin, and fengycin [3]; phospholipids such as phosphatidylcholine and phoshatidylethanolamine [4]; and polymeric biosurfactants including emulsan, liposan, lipomanan, and alasan [5]. Rhamnolipids (RLs) are the most studied biosurfactants that are currently available from Evonik, the world’s first commercial-scale facility for these biosurfactants (https://corporate.evonik.com/, accessed on 18 January 2014). However, most other biosurfactants are not commercially available in large quantities. Limited types and quantities impede the academic and industrial applications.

A mixture of phosphorus-free ornithine lipids (OLs) is found in the culture supernatant of *Myroides* sp. SM1, which is capable of emulsifying crude oil [6]. OLs appear to be widely distributed in eubacteria, but not in eukaryotes and archaea [7]. A 3-hydroxy fatty acyl group is attached in amide linkage to the α-amino group of ornithine in OLs, while a second fatty acyl group is ester-linked to the 3-hydroxy position of the first fatty acid [8,9]. OL biosynthesis is a two-step reaction: the first step is the formation of lyso-ornithine lipid (LOL) catalyzed by *N*-acyltransferase OlsB in presence of 3-hydroxyacyl-AcpP and ornithine [10]. The second step is the formation of OL catalyzed by *O*-acyltransferase OlsA using acyl-acyl carrier protein (acyl-AcpP) as an acyl donor in the acylation of LOL, a precursor of OL [11].

LOL has been obtained in a screening for biosurfactants derived from uncultivable microbes using cloned environmental DNA (eDNA) that are expressed in *Pseudomonas putida* [12]. Additionally, Kristoffersen et. al. [13] reported two novel LOLs isolated from an arctic marine *Lacinutrix sp.* bacterium. Hence, LOL could be a useful emulsifier for oil recovery like RL. However, conditions for the production of LOL in microbial culture supernatant in a measurable level (e.g., >1 mg L^−1^) for academic and industrial studies are not available.

To explore the possibility of producing LOL in microbial culture supernatant at a level suitable for academic and industrial studies, we have chosen a strong version of arabinose regulatable araC-P_BAD-SD_ promoter [14] to control the heterologous expression of *rhlAB* and *olsB* in a non-pathogenic *P. putida* KT2440 strain for production of mRL and LOL, respectively. In this study, we were able to produce mRL at a maximum level of 1.5 g L^−1^ in the supernatant of the *KT2440/pOEs-rhlAB* cultivated with 0.02% arabinose supplementation. With the same setting, we show that we were also capable of producing LOL at a maximum level of 10 mg L^−1^ in the supernatant of the *KT2440/pOEs-olsB* culture supplemented with 0.2% arabinose. Significantly, we found that the capacity to emulsify crude oil by LOL was four times higher than by mRL, implying that LOL can be applied for enhanced oil recovery. We propose that this araC-P_BAD-SD_ regulatable system controlling the heterologous DNA expression in *P. putida* KT2440 could be a useful tool for production of RL, LOL, and possibly other biosurfactants.

## 2. Results

### 2.1. Different Expression Patterns of luxCDABE Controlled by P_BAD_ or P_BAD-SD_ between Escherichia coli and Pseudomonas aeruginosa PAO1

We wanted to investigate the levels of heterologous gene expression controlled by the P_BAD_ promoter and the strong-expression version of the P_BAD-SD_ promoter in which an additional ribosome-binding site was added [14]. To monitor the expression level in real-time, an operon *luxCDABE* sequence derived from *Aliivibrio fischeri* [15] was cloned under the control of P_BAD_ and P_BAD-SD_ promoters in the multi-host vector pBBR1MCS5 [16] to yield the pOE-luxCDABE (or pOE-lux) and pOEs-lux plasmids, respectively (Table 1) (Section 4). To test the luminescence production, the resulting two plasmids were separately transformed into *E. coli* K12 and *P. aeruginosa* PAO1 (see Section 4). This is because *E. coli* is the most frequently used host for heterologous gene expression [17], and *P. aeruginosa* is the best rhamnolipid producer [18].

**Figure 1:**
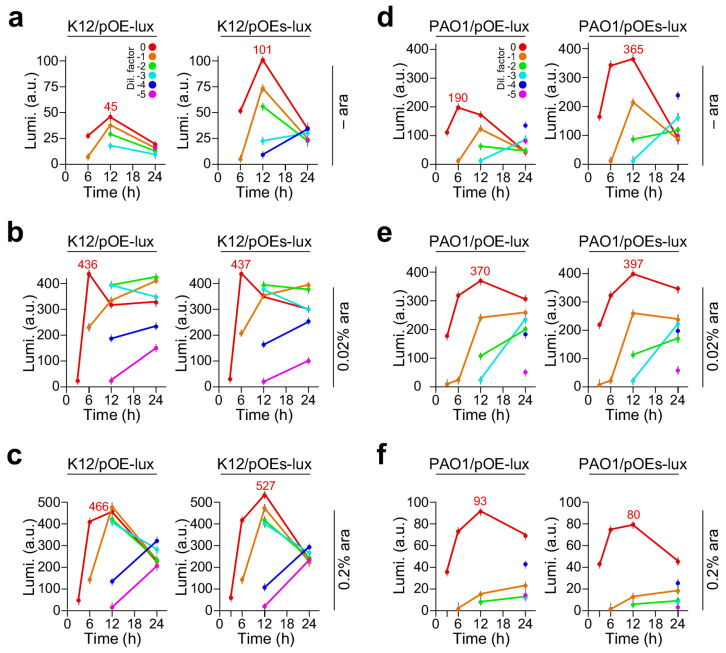
Luminescence produced in *E*. *coli* K12 and *P. aeruginosa* PAO1 on LB plates supplemented with 0, 0.02%, and 0.2% arabinose. X- and Y-axes indicate the time (h) and luminescence level (arbitrary units, a. u.) of the cultures with various dilution factors indicated by different colors. A color key for dilution factors is shown in panels a and d. Luminescence expression is controlled by P_BAD_ (pOE-lux) and P_BAD-SD_ (pOEs-lux) promoters in the left and right panels, respectively. (**a**) Luminescence produced by *E. coli K12(pOE-lux)* (left panel) and *K12(pOEs-lux)* (right panel) on LB plate supplemented without arabinose (– ara), and (**b**) supplemented with 0.02% arabinose and (**c**) with 0.2% arabinose. (**d**) *P. aeruginosa PAO1/pOE-lux* and *PAO1/pOEs-lux* strains growing on LB plates with arabinose supplementation of 0, (**e**) 0.02%, and (**f**) 0.2% concentrations.

Cultures of the *K12/pOE-lux* and *K12/pOEs-lux* were ten-fold diluted and spotted on to LB plates supplemented with 0%, 0.02%, and 0.2% arabinose (Figure S1A). Luminescence produced by the cells on plates was measured with a luminescence detector (Tenan BioScience Pte Ltd., Shanghai, China) exposing for 10 s at 3 h, 6 h, 12 h, and 24 h after incubation at 30 °C (Figure S1B). Average levels of luminescence produced by K12/pOE-lux and K12/pOEs-lux strains at various time points were plotted for each diluted cell spot (Figure 1a–c).

As a control, strain *K12/pOE* containing a blank plasmid grew normally but exhibited no luminescence with or without arabinose supplementation (see Figure S1A,B, top two rows in plate). However, luminescence was detected in *K12/pOE-lux* and *K12/pOEs-lux* strains even without arabinose supplementation (Figure 1a). Notably, the maximum level of luminescence in *K12/pOEs-lux* without arabinose induction was 2.2-fold higher than that of *K12/pOE-lux* based on the undiluted culture or with a dilution factor of zero (101 ± 5.1 vs. 45 ± 2.4, *p*-value = 2.0 × 10^−3^, n = 3). In the case of arabinose supplementation at concentrations of 0.02% and 0.2% (Figure 1b,c), we found that the maximum luminescence levels in the undiluted culture were not changed much (i.e., level change ≤ 1.2-fold) between strains *K12/pOEs-lux* and *K12/pOE-lux*. Nevertheless, the luminescence levels with the 0.2% arabinose supplementation were slightly higher than those with 0.02% arabinose.

Similarly, the maximum luminescence levels produced by *PAO1/pOEs-lux* without arabinose supplementation were 1.9-fold higher than that of *PAO1/pOE-lux* (365 ± 12.1 vs. 190 ± 10.9, *p*-value = 9.9 × 10^−3^, n = 3) (Figure 1d and Figure S2). Notably, we found that the maximum luminescence levels produced by *PAO1/pOEs-lux* and *PAO1/pOE-lux* cells supplemented with 0.02% arabinose were significantly higher than that of 0.2% arabinose (pOE-lux, 370 ± 11.3 vs. 93 ± 10.8, *p*-value = 5.3 × 10^−3^, n = 3; pOEs-lux, 397 ± 13.2 vs. 80 ± 10.1, *p*-value = 7.8 × 10^−3^, n = 3) (Figure 1e,f). These results suggest that the level of expression controlled by P_BAD-SD_ promoter in pOEs was not effectively repressed in the absence of the inducer. Additionally, induction condition of the same construct behaved differently in *E. coli* K12 and *P. aeruginosa* PAO1.

### 2.2. Expression Pattern of luxCDABE Controlled by PBAD or PBAD-SD in P. putida

**Figure 2:**
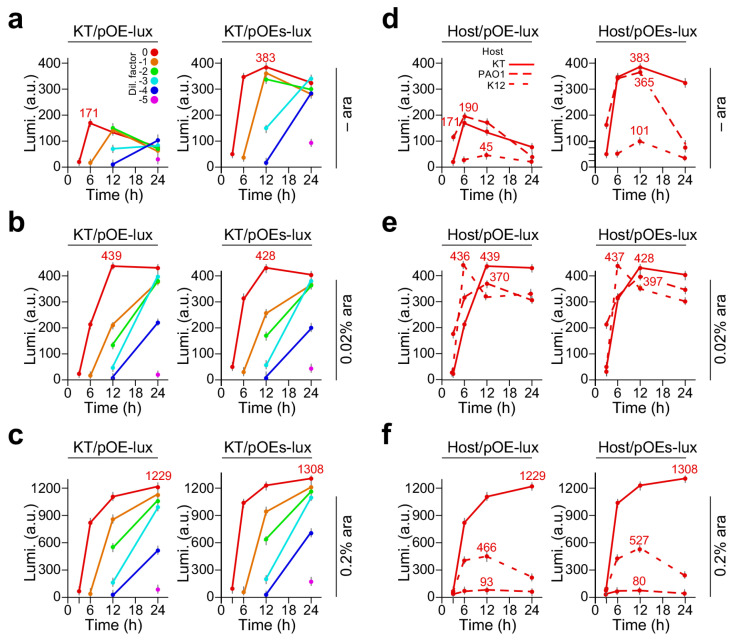
Luminescence produced by *P. putida KT2440/pOE-lux* and *KT2440/pOEs-lux* on LB plates supplemented with 0, 0.02%, and 0.2% arabinose. X- and Y-axes indicate the time (h) and luminescence level (a. u.) of the cultures with various dilution factors. A color key for dilution factors is shown in panel a. Luminescence expression is controlled by P_BAD_ (pOE-lux) and P_BAD-SD_ (pOEs-lux) promoters in the left and right panels, respectively. (**a**) Luminescence produced by *P. putida KT2440/pOE-lux* (left panel) and *KT2440/pOEs-lux* (right panel) on LB plates with arabinose supplementation at 0, (**b**) at 0.02%, and (**c**) at 0.2% concentrations. (**d**) Luminescence produced by pOE-lux (left panel) and pOEs-lux (right panel) bearing strains of *E. coli* K12, *P. aeruginosa* PAO1, and *P. putida* KT2440 without dilution on LB plate with arabinose supplementation of 0, (**e**) 0.02%, and (**f**) 0.2% concentrations. Line key for host cells is shown in panel (**d**).

We subsequently examined the heterologous expression of *luxCDABE* in *P. putida* KT2440, a non-pathogenic strain suitable for large-scale production of bioactive molecules [19]. To this end, we found that the leaky expression levels by the pOEs-lux were 2.24-fold higher than that of pOE-lux (383 ± 11.1 vs. 171 ± 7.9, *p*-value = 8.2 × 10^−3^, n = 3) (Figure 2a), similar to the observation in *E. coli* and *P. aeruginosa* (see Figure 1a,d). We found that with 0.02% arabinose supplementation, the levels of luminescence in *KT/pOE-lux* were significantly increased compared to that without arabinose supplementation (level change = 2.6-fold, *p*-value = 6.8 × 10^−4^, n = 3) (Figure 2b). However, the luminescence levels in *KT/pOE-lux* and *KT/pOEs-lux* with 0.02% arabinose supplementation were similar (level change = 1.1, *p*-value = 0.06, n = 3).

**Table 1:** Oligonucleotides, plasmids, and strains used in this study.

(A) Oligonucleotides		
Name	Fragment	Sequence (5′-3′)
OE-lux-F	P_BAD_ (for lux)	cgctctagaactagtggatccTTATGACAACTTGACGGCTACATCA
OE-lux-R		aagcctgaattccccggatccCCAAAAAAACGGGTATGGAGAA
OEs-lux-F	P_BAD-SD_ (for lux)	cgctctagaactagtggatccTTATGACAACTTGACGGCTACATCA
OEs-lix-R		aagcctgaattccccggatccAATTGCAATCGCCATCGTTT
OEs-rl-F1	P_BAD-SD_ (for rhlAB)	cgctctagaactagtggatccTTATGACAACTTGACGGCTACATCA
OEs-rl-R1		tcgcgccgcatAATTGCAATCGCCATCGTTT
OEs-rl-F2	rhlAB	attgcaattATGCGGCGCGAAAGTCTGTT
OEs-rl-R2		cttgatatcgaattcctgcagCTCCGTCATTCCTCATTGCAGTAAG
OEs-lol-F1	P_BAD-SD_ (for olsB)	same as OEs-rl-F1
OEs-lol-R1		cggtctgggtcatAATTGCAATCGCCATCGTTT
OEs-lol-F2	olsB	tgcaattATGACCCAGACCGCCATTACC
OEs-lol-R2		tccagcagctggataTCAGACCGCTGCCTTGAAGT
**(B) Plasmid**		
**Name**	**Usage or relevant genotype**	**Reference**
pGEN-luxCDABE	For luxCDABE amplification	[15]
pBAD18	For P_BAD_ amplification	[14]
pBAD18s	For P_BAD-SD_ amplification	[14]
pBBR1MCS-5	Shuttle vector	[16]
pOE-lux	pBBR1MCS5-P_BAD_-luxCDABE	This study
pOEs-lux	pBBR1MCS5-P_BAD-SD_-luxCDABE	This study
pOEs-rhlAB	pBBR1MCS5-P_BAD-SD_-rhlAB_ZS1_	This study
pOEs-olsB	pBBR1MCS5-P_BAD-SD_-olsB_PAO1_	This study
**(C) Strains**		
**Name**	**Relevant genotype**	**Reference**
K12	Wild-type *E. coli* K12	BioSciBio
PAO1	Wile-type *P. aeruginosa* PAO1	BioSciBio
KT2440	Wild-type *P. putida* KT2440	BioSciBio
ZS1	Wild-type *P. aeruginosa* ZS1	[20]
K12(pOE-lux)	K12(P_BAD_-luxCDABE)	This study
K12(pOEs-lux)	K12(P_BAD-SD_-luxCDABE)	This study
PAO1/pOE-lux	PAO1/P_BAD_-luxCDABE	This study
PAO1/pOEs-lux	PAO1/P_BAD-SD_-luxCDABE	This study
KT/pOE-lux	KT2440/P_BAD_-luxCDABE	This study
KT/pOEs-lux	KT2440/P_BAD-SD_-luxCDABE	This study
KT/pOEs-rhlAB	KT2440/P_BAD-SD_-rhlAB_ZS1_	This study
KT/pOEs-olsB	KT2440/P_BAD-SD_-olsB_PAO1_	This study

When arabinose supplementation was increased to 0.2%, the exposure time of 10 s for determining the luminescence levels exceeded the saturation point (Figure S3). After adjusting the exposure time to 3 s, we found that the luminescence levels produced by KT/pOEs-lux and KT/pOEs-lux with supplementation of 0.2% arabinose were increased by 2.8-fold (*p*-value = 7.1 × 10^−4^, n = 3) and 3.1-fold (*p*-value = 7.4 × 10^−4^, n = 3) compared to that with 0.02% arabinose supplementation, respectively (Figure 2c). This result indicated that *P. putida* KT2440 was a useful strain for robust expression of heterologous DNA such as *luxCDABE*.

To compare expression levels of *luxCDABE* under the control of P_BAD_ and P_BAD-SD_ promoters on LB plates with supplementation of 0, 0.02%, and 0.2% arabinose concentrations, we chose the undiluted cells that contained the lowest number of missing measurements along the time course experiment compared to that of diluted cells. Leaky expression levels (i.e., no arabinose inducer) produced by various cells with P_BAD-SD_ promoter-controlled *luxCDABE* appeared to be twice as high as that with P_BAD_ promoter (Figure 2d). Under subsaturation induction (i.e., 0.02% arabinose supplementation), all strains showed expression levels between 300 and 450 arbitrary units (a.u.) with P_BAD_ or P_BAD-SD_ promoters 12 h after induction (Figure 2e). However, under saturation induction (i.e., 0.2% arabinose supplementation), differences in expression levels between strains were apparent: *P. putida* showed the highest levels (i.e., between 1229 a.u. and 1308 a.u.) with either P_BAD_ or P_BAD-SD_ promoter (Figure 2f) among the three strains. *E. coli* exhibited the lowest expression level (i.e., between 80 a.u. and 93 a.u.) regardless of P_BAD_ or P_BAD-SD_ promoter. *P. aeruginosa* PAO1 displayed the expression levels between those of *P. putida* and *E. coli* (i.e., between 466 a.u. and 527 a.u.). These results indicate that the expression regulation of P_BAD_- or P_BAD-SD_-controlled *luxCDABE* is a complex. While *P. aeruginosa* showed higher expression levels under subsaturation induction than under saturation induction, *P. putida* exhibited higher expression levels under saturation induction than under subsaturation induction.

### 2.3. Optimal Level of Rhamnolipid Production by P. putida KT2440/pOEs-rhlAB with Supplementation of Subsaturation 0.02% Arabinose

We wanted to investigate the rhamnolipid (RL) production by heterologous expression of the P_BAD-SD_ promoter-controlled *rhlAB_ZS1_* genes in *P. putida* KT2440. The sequence of *rhlAB_ZS1_* genes was derived from *P. aeruginosa* ZS1 capable of producing RL to a level of 30 g L^−1^ when cultivated in mineral salt (MS) medium supplemented with 2% glucose or glycerol as the sole carbon source in a shake flask [20]. Hence, *rhlAB_ZS1_* DNA sequences were PCR-amplified from the ZS1 genome and sub-cloned in pBBR1MCS-5 vector [16] under the control of P_BAD-SD_ promoter to generate the pOEs-rhlAB_ZS1_ plasmid (see Section 4). The resulting plasmid was electroporated into *P. putida* KT2440 to produce the *KT2440/pOEs-rhlAB* strain.

**Figure 3:**
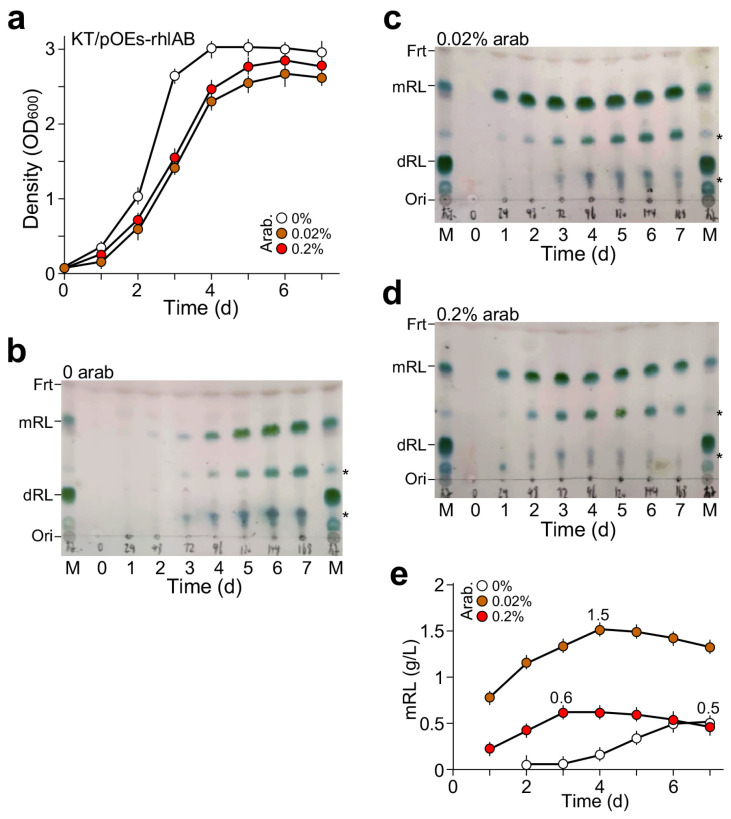
mRL produced by *P. putida KT2440/pOEs-rhlAB* in MS medium plus 2% glycerol as the sole carbon source with supplementation of arabinose at 0, 0.02%, and 0.2%. (**a**) Growth curve analysis of the *KT2440/pOEs-rhlAB* in medium supplemented with or without arabinose. X- and Y-axes indicate time (d) and cell density (OD value at 600 nm wavelength), respectively. (**b**) TLC analysis of mRL produced by *KT2440/pOEs-rhlAB* without arabinose supplementation, (**c**) with 0.02% arabinose, and (**d**) with 0.2% arabinose supplementation. Asterisk (*) indicates the contaminants. (**e**) Semi-quantitation of mRL produced by *KT2440/pOEs-rhlAB* in medium supplemented with various amounts of arabinose based on TLC analysis shown in (**b**–**d**).

MS medium plus 2% glycerol as the sole carbon source was used to cultivate the *KT2440/pOEs-rhlAB* strain with supplementation of arabinose at concentrations of 0, 0.02%, and 0.2%. Growth curve analysis indicated that without arabinose supplementation, the cells entered the onset of stationary phase three days after growth (Figure 3a). On the other hand, the onset of the stationary phase in cultures supplemented with 0.2% or 0.02% arabinose occurred four days after growth, one day later compared to the culture without arabinose supplementation. Slow growth rate and low maximum cell density would mean that the culture accumulated more mRL and less cell biomass.

Rhamnolipid produced in cultures with or without arabinose supplementation was assayed on TLC plates after ethyl acetate extraction from cell-free supernatant at various time points during cell growth (see Section 4). We found that *KT2440/pOEs-rhlAB* without arabinose supplementation could produce mono-rhamnolipid (mRL) with a peak level seven days after growth (Figure 3b). Notably, the level of mRL production in culture with 0.02% arabinose supplementation was higher than that of 0.2% arabinose supplementation (Figure 3c,d). Semi-quantitative estimation of mRL contents based on TLC analysis showed that the maximum level in the culture with 0.02% arabinose supplementation was 2.5-fold higher than that with 0.2% arabinose supplementation (1.5 g L^−1^ vs. 0.6 g L^−1^, *p*-value = 8.9 × 10^−4^, n = 3) (Figure 3e). Hence, the maximum production and productivity of mRL by *KT2440/pOEs-rhlAB* with 0.02% arabinose supplementation were 1.5 g L^−1^ and 0.38 g d^−1^ L^−1^, respectively.

### 2.4. P. putida KT2440/pOE-rhlAB_ZS1_ Produces mRL Exhibiting Similar Types of Congeners and Their Relative Levels as Those Found in ZS1

**Figure 4:**
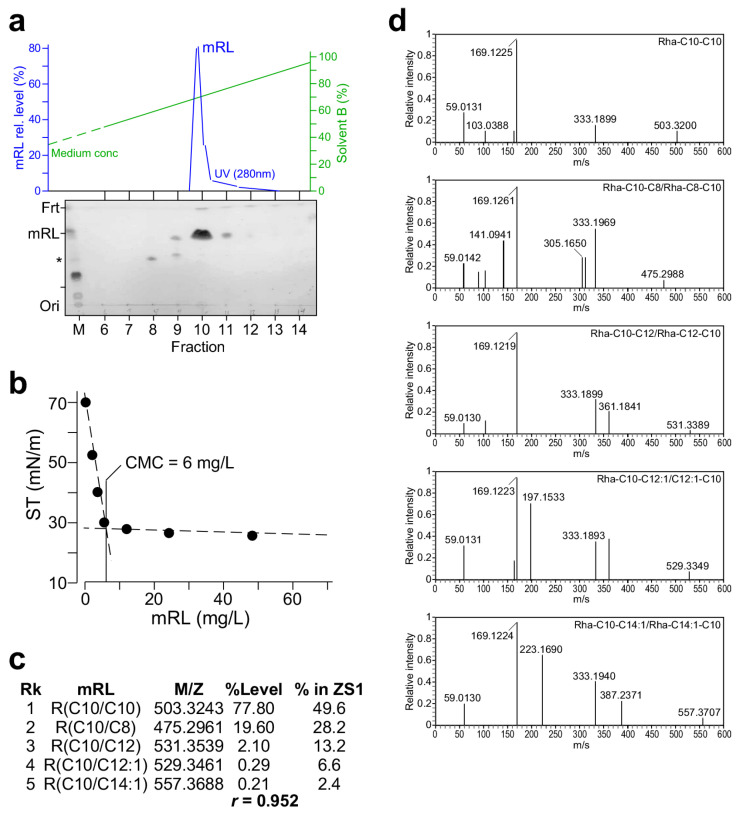
Characterization of the mRL produced by *KT2440/pOEs-rhlAB*. (**a**) Medium-pressure liquid chromatographic purification of mRL raw extract derived from the cell-free supernatant of the *KT2440/pOEs-rhlAB* culture. Top panel shows the mRL peak eluted from a C8 column. Bottom panel shows the TLC analysis of various fractions derived from elution. (**b**) Critical micelle concentration (CMC) analysis. X- and Y-axis indicate mRL concentration (mg L^−1^) and surface tension (ST; mN m^−1^). MPLC-purified mRL is two-fold diluted and assayed for surface tension. (**c**) mRL congeners and their relative level revealed by LC-MS/MS analysis. (**d**) MS2 spectra of various mRL congeners detected in the purified mRL.

It is known that *P. aeruginosa* ZS1 produces 11 di-rhamnolipid (dRL) congeners and seven mono-rhamnolipid (mRL) congeners [20]. In this study, the *rhlAB*, but not *rhlC*, gene was transformed into *P. putida* for the production of mRL (see Figure 3). To investigate whether types of mRL congener molecules and their relative levels produced by *KT2440/pOEs-rhlAB_ZS1_* resembled those found in ZS1, the mRL in the cell-free supernatant of *KT2440/pOEs-rhlAB_ZS1_* culture was first extracted using the ethyl acetate solution. The resulting raw material was subsequently dissolved in methanol and subjected to purification using medium-pressure liquid chromatography (AKTA, Cytiva, Uppsala, Sweden). A single peak was eluted at the concentrations of elution buffer between 65% and 70% acetonitrile (Figure 4a, upper panel). TLC analysis of various fractions showed that the peak fraction contained most of the mRL (Figure 4a, lower panel). Consistent with this, the peak fraction displayed the highest oil-spreading activity among others (Figure S4).

Critical micelle concentration (CMC) of mRL was determined using the AKTA-eluted peak fraction that was dried and dissolved in methanol at a concentration of 10 mg mL^−1^. By using a surface tensiometer (BZY-B tensiometer, Fangrui Instrument Co. Ltd., Shanghai, China) with a du Nouy ring, we showed that the CMC of the KATA-purified mRL was 6 mg L^−1^ (Figure 4b).

LC-MS/MS analysis (see Section 4) of the AKTA-purified mRL revealed five congener molecules and their relative abundances (Figure 4c,d). These five mRL congeners produced by *KT2440/pOEs-rhlAB_ZS1_* were the top-ranked mRL in ZS1. Furthermore, their relative abundances of the mRL congener molecules were correlated with a coefficient of 0.952 (see Figure 4c). These results suggest that biosynthesis of RL congener molecules is largely determined by the sequence of *rhlAB* genes, consistent with observations reported by others [21,22,23].

### 2.5. Optimal Level of Lyso-Ornithine Lipid Production by P. putida KT2440/pOEs-olsB with Saturation Induction of 0.2% Arabinose Supplementation

LOL is a novel biosurfactant with many potential applications [24]. To explore the LOL production condition, *pa4350|olsB* sequence was PCR-amplified from the genomic DNA derived from *P. aeruginosa* PAO1 [25] and cloned under the control of P_BAD-SD_ promoter in pBBR1MCS-5 vector (see Section 4, Methods).

**Figure 5:**
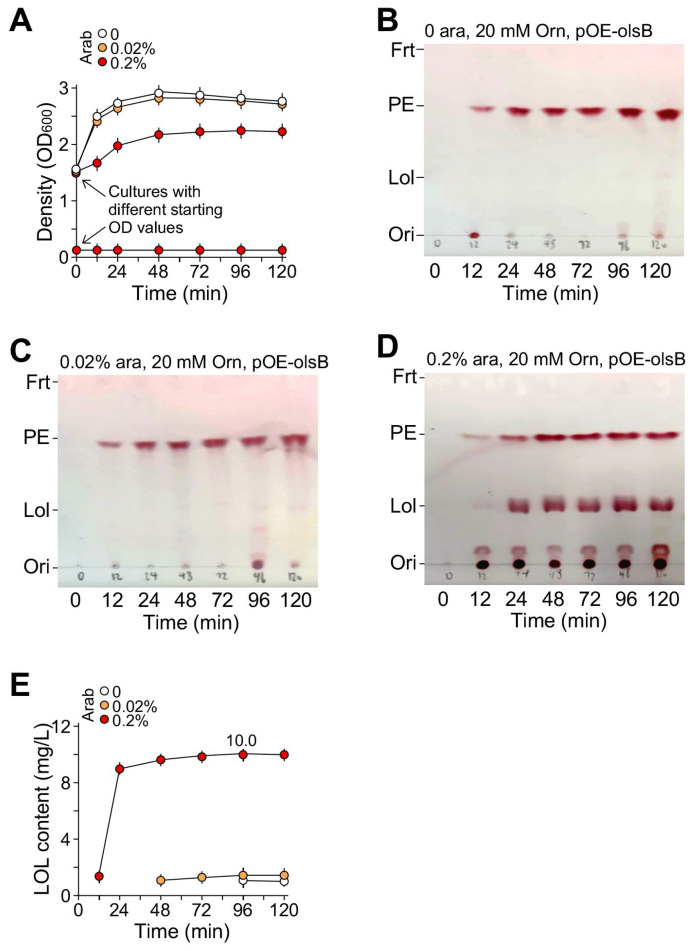
LOL produced by *P. putida KT2440/pOEs-olsB* in MS plus 3% acetate and 20 mM ornithine medium with supplementation of arabinose at 0, 0.02%, and 0.2%. (**A**) Growth curve analysis of the *KT2440/pOEs-olsB* in medium supplemented with or without arabinose or with different starting OD_600_ values for 0.2% arabinose supplementation. X- and Y-axes indicate time (h) and cell density (OD value at 600 nm wavelength). (**B**) TLC analysis of LOL produced by *KT2440/pOEs-olsB* with 0% arabinose, (**C**) with 0.02% arabinose, and (**D**) with 0.2% arabinose supplementation. Ori, LOL, PE, and Frt stand for origin, lyso-ornithine lipid, phosphatidylethanolamine, and front. (**E**) Semi-quantitation of LOL produced by *KT2440/pOEs-olsB* in medium supplemented with various amounts of arabinose based on TLC analysis shown in (**B**–**D**).

We found that *KT2440/pOEs-olsB* was unable to grow in high-nutrient medium, that is, in the 2× (double strength) MS medium plus 3% NaAc as the sole carbon source and 20 mM ornithine when the OD_600_ value of the starting culture was 0.2 or less. Hence, we increased the OD_600_ value of the starting culture to 1.5 for LOL production. As a result, the cultures entered the onset of the stationary phase one day after growth (Figure 5a). On the other hand, the high starting OD_600_ value of 1.5 in normal nutrient medium like MS + 2% NaAc failed to produce surfactant activity.

Supernatant of the culture was taken and extracted by the methanol–chloroform protocol (see Methods). TLC analysis of the supernatant indicated the presence of membrane lipid phosphatidylethanolamine (PE) that might be derived from the lysed cells. While *KT2440/pOEs-olsB* growth under the conditions with 0 or 0.02% arabinose supplementation showed no apparent production of LOL (Figure 5b,c), cell growth with the supplementation of 0.2% arabinose clearly showed production of LOL (Figure 5d). The semi-quantitation of LOL based on the staining of TLC plate using ninhydrin (2,2-dihydroxyindane-1,3-dione) (see Section 4) indicated maximum LOL production of 10 mg L^−1^ (Figure 5e). The maximum productivity of LOL was 7.5 mg d^−1^ L^−1^. We showed that the production of LOL in KT2440 was dependent on pOEs-olsB plasmid but not ornithine supplementation (Supplementary Materials: Figure S5).

### 2.6. Lipid Moiety of the Major LOL Congener Molecules Resembles That of Membrane Lipids

**Figure 6:**
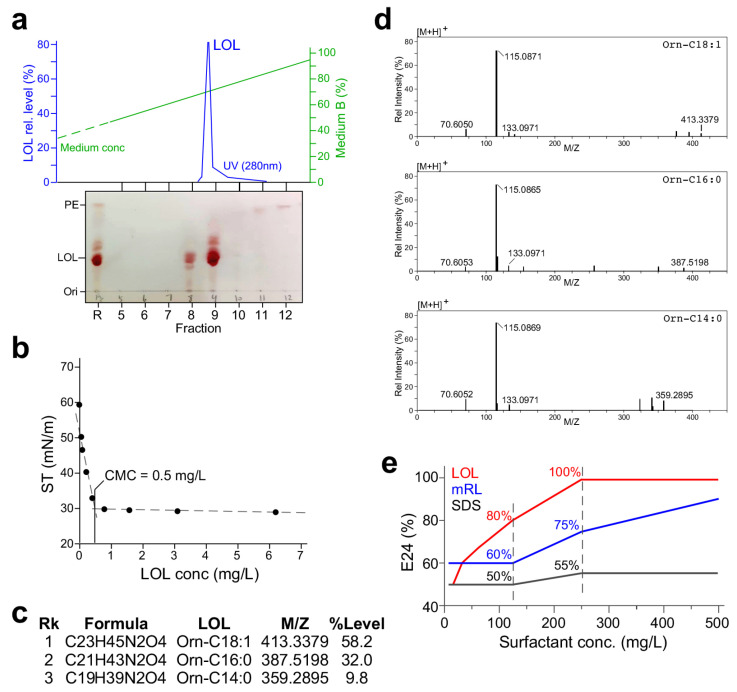
Characterization of the LOL produced by *KT2440/pOEs-olsB*. (**a**) Medium-pressure liquid chromatographic purification of LOL raw extract derived from the cell-free supernatant of the *KT2440/pOEs-olsB* culture. Top panel shows the mRL peak eluted using a C8 column. Bottom panel shows the TLC analysis of various fractions derived from elution. (**b**) Critical micelle concentration (CMC) analysis. X- and *Y*-axis indicate LOL concentration (mg L^−1^) and surface tension (mN m^−1^). MPLC-purified LOL is two-fold diluted and assayed for surface tension. (**c**) LOL congeners and their relative levels revealed by LC-MS/MS analysis. (**d**) MS2 spectra of various LOL congeners detected in the purified mRL. Fragments of 70, 115, and 133 *m*/*z* are signatures of lyso-ornithine lipid. (**e**) Emulsification index E24% of LOL, mRL, and SDS.

LOL purified through medium-pressure liquid chromatography would facilitate the identification of LOL structures using LC-MS/MS methodology. Hence, LOL raw extract derived from supernatant of *KT2440/pOEs-olsB* cultures using the methanol–chloroform method was dissolved in methanol and loaded on to the AKTA C8 reverse phase column (see Section 4, Methods). LOL was eluted at a concentration of 70% acetonitrile elution buffer (Figure 6a, upper panel). That was consistent with the TLC analysis and oil-spreading assay of the eluent fractions (Figure 6a, bottom panel; Figure S6).

The AKTA-purified LOL was dissolved in methanol to a concentration of 1 mg L^−1^, and its two-fold dilutions were assayed for surface tension. Based on the surface tension of various diluents, we concluded that the CMC of the AKTA-purified LOL was 0.5 mg L^−1^ (Figure 6b), 12-fold lower than that of mRL. LC-MS/MS analysis of the AKTA-purified LOL revealed three congener molecules and their relative levels: 58.2% of orn-C18:1, 32.0% of orn-C16:0, and 9.8% of orn-C14:0. The lipid moiety of 90% LOL was C18:1 and C16:0, which were the major lipid moieties of the membrane lipid in *Pseudomonas* [26]. MS2 spectra of the three LOL congener molecules contained the ornithine lipid signature mass species of 70 *m*/*z*, 115 *m*/*z*, and 133 *m*/*z* (Figure 6d). Emulsification capacity of the biosurfactants LOL, mRL, and synthetic surfactant sodium dodecyl sulfate (SDS) mixed with an equal volume of crude oil was analyzed (see Section 4). At a concentration of 125 mg L^−1^, we found that E24 values of LOL, mRL, and SDS were 80%, 60%, and 50%, respectively (Figure 6e). When the surfactant concentration was raised to 250 mg L^−1^, E24 values of LOL, mRL, and SDS were 100%, 75%, and 55%, respectively. These results indicated that emulsification capacity of LOL was the highest among the three surfactants. Importantly, we showed that the emulsification capacity of LOL was four times higher than that of mRL.

## 3. Discussion

In this study, we show that the novel biosurfactant LOL can be produced at a level of 10 mg L^−1^ in the non-pathogenic strain *P. putida* KT2440 by using heterologous expression of a strong arabinose regulatable promoter P_BAD-SD_-controlled *olsB* gene (see Figure 5). With the same system, it produces mRL at a maximum level of 1.5 g L^−1^ (see Figure 3), which is 150-fold higher than that of LOL. It is is most likely that dTDP-rhamnose is highly abundant primarily for biosynthesis of lipopolysaccharide (LPS) in *P. putida* [27]. Given that increased ornithine supplementation to 40 mM does not increase the LOL yield compared to that of 20 mM ornithine supplementation, we propose that a feedback regulation of LOL biosynthesis exists. Omics-based analysis should be able to identify candidate regulators that can be altered by metabolic engineering to further enhance the LOL yield when ornithine supplementation increases.

Although the P_BAD_ promoter is a widely used regulatable expression system in microbes, it has limitations. At intermediate levels of gene expression by subsaturating induction, individual cells of the culture are non-uniform [28]. In addition, P_BAD_ promoter-controlled gene expression by saturating induction is low compared to that of subsaturating induction [29].

This may explain why luminescence levels produced by P_BAD_ or P_BAD-SD_ promoter-controlled *luxCDABE* in *P. aeruginosa* PAO1 with subsaturating 0.02% arabinose induction are four-fold higher than that with saturating induction of 0.2% arabinose (see Figure 1e,f). However, the same construct in *P. putida* KT2440 exhibits high expression levels by saturating induction (see Figure 2). This result indicates that expression levels controlled by P_BAD_ promoter under saturating induction may differ from strain to strain.

Additionally, we show that maximum level of RL production in *KT2440/pOEs-rhlAB* is achieved with subsaturating induction of 0.02% arabinose (see Figure 3). There have been a number of reports on heterologous production of mRL in *P. putida* KT2440. By applying IPTG regulatable promoter P_tac_-controlled expression of *rhlAB* in *P. putida* KT2440 cultivated in M9 medium plus vegetable oil as sole carbon source, Setoodeh et al. [30] managed to produce 0.57 g L^−1^ mRL. After *phaC* was knocked out, P_tac_-rhlAB expression in KT2440 growing LB with glucose as the sole carbon source, Wittgens et al. [31] were able to produce mRL at a level of 1.5 g L^−1^. Tiso et al. [32] reported that salicylate-inducible promoter NagR/PnagAa-controlled expression of *rhlAB* achieved 1.5 g L^−1^ mRL production in KT2440. They found that RL production with subsaturation induction was higher than that of saturation induction [33]. This was similar to what we found in mRL using P_BAD-SD_-controlled *rhlAB* expression in this study (see Figure 3).

On the other hand, maximum level of LOL production in *KT2440/pOEs-olsB* requires saturating induction of 0.2% arabinose (see Figure 5). This result suggests that induction for maximum expression level of genes controlled by P_BAD_ promoter may be affected by promoter-controlled gene sequences. Hence, to obtain the maximum expression or production levels, it is advisable to test experimentally for optimal induction conditions when the regulatable P_BAD_ promoter system in *P. putida* is utilized.

Although there is no other report on LOL production in *P. putida* KT2440 for comparison, studies on the production of *N*-acyl amino acid began to appear after the isolation and identification of enzyme sequences responsible for its biosynthesis [34]. By using P_T7_-controlled *N*-acetyltransferase expression in *E. coli*, Cho et al. [35] were able to produce *N*-acyl-ornithine and *N*-acyl lysine to a level of 1 mg L^−1^. To circumvent the low yield, Haeger et al. [36] utilized the purified enzyme to catalyze the synthesis of *N*-acyl amino acid to a conversion rate up to 75%. It would be interesting to test the P_BAD-SD_ expression system for the production of *N*-acyl amino acids in KT2440 in future.

Removal of hydrocarbons involves several mechanisms [37], including emulsification that packs oil into small droplets suspended in the water solution. RL has shown its potential in this aspect compared to synthetic surfactants. Ramirez et al. studied the performance of RL in recovering oil sludge compared with other commonly used surfactants such as Triton X-100 and X-114, Tween 80, and SDS. According to their study, RL had one of the highest oil-recovery rates, around 40–70% [38]. We have previously shown that the minimum concentration of RL (mRL + dRL) derived from *P. aeruginosa* ZS1 is 1.2 g L^−1^ to reach the emulsification index E24 value of 100% when mixed with an equal volume of crude oil, while SDS at a concentration of 10 g L^−1^ only yields an E24 value of 75% [20]. In this study, we have shown that a concentration of 1 g L^−1^ mRL produced from *KT2440/pOE-rhlAB* is needed to reach an E24 value of 100% when mixed with crude oil (see Figure 6e). Nevertheless, LOL at a concentration of 0.25 g L^−1^ derived from *KT2440/pOE-olsB* is sufficient to produce an E24 value of 100%, four times more efficient in terms of emulsifying crude oil than mRL. Hence, LOL will be a good addition to RL for environmental remediation.

In conclusion, we have shown in this study that using P_BAD-SD_-regulatable systems for heterologous expression in *P. putida* is promising for production of biosurfactants such as RL and LOL. Moreover, we have shown that LOL has a higher emulsification capacity than mRL and propose that LOL is suitable for environmental remediation.

## 4. Materials and Methods

### 4.1. Strains and Culture Manipulation

*E. coli* K12 (Cat# PD098), *P. aeruginosa* PAO1 (Cat# V0091), and *P. putida* KT2440 (Cat# PD062) were purchased from BioSciBio (Hangzhou, China) (Table 1). *P. aeruginosa* ZS1 was isolated from oil sludge [20]. Various media such as rich medium LB (LB, 1 L contains: tryptone 10 g, Yeast Extract 5 g, NaCl 10 g) [39] and minimum mineral (MS) medium (MS, 1 L contains: 0.6 g Na_2_HPO_4_, 0.2 g KH_2_PO_4_, 4.0 g NaNO_3_, 0.3 g MgSO_4_, 0.01 g CaCl_2_, 0.01 g FeSO_4_) [40] were used in this study. Arabinose inducible promoters P_BAD_ and P_BAD-SD_ [14] were chosen to control the expression of heterologous genes or protein-coding sequences without 5′-UTR such as *luxCDABE* [15], *rhlAB* [20], and *olsB* [25] in *P. putida* KT2440. For RL production, KT2440/pOEs-rhlAB was cultivated in MS medium containing 2% glycerol as the sole carbon source. On the other hand, for LOL production, *KT2440/pOEs-olsB* was cultivated in 2 × MS medium containing 3% NaAc.

### 4.2. Plasmid Construction

Arabinose regulable promoter-controlled expression of genes *luxCDABE*, *rhlAB*, and *olsB* was constructed using the pBBR1MCS-5 plasmid [8]. *luxCDABE* fragment was obtained via *Bam*HI and *Pst*I double digestion of pGEN-luxCDABE plasmid [15]. The resulting fragment was cloned in the pBBR1MCS-5 at the *Bam*HI and *Pst*I sites. Subsequently, the P_BAD_ and P_BAD-SD_ PCR amplified fragments were inserted at the *Bam*HI site, the 5′ end of the *luxCDABE* sequence using a Vazyme recombinase cloning kit (Vazyme, Nanjing, China) to produce the plasmid pOE-luxCDABE and pOEs-luxCDABE. For plasmid pOEs-rhlAB and pOEs-olsB plasmids, the promoter P_BAD-SD_ sequence was PCR-amplified using pBAD18 as template. The *rhlAB* (or *olsB*) sequences were amplified using ZS1 (or PAO1) genomic DNA. *Bam*HI-digested pBBR1MCS-5 plasmid DNA was then ligated with P_BAD-SD_ and *rhlAB* in multiple fragments to generate pOEs-rhlAB plasmid using a recombinase Vazyme cloning kit (Vazyme). Similarly, the *olsB* fragment was used for generation of pOEs-olsB plasmid. All primer sequences used in plasmid construction are shown in Table 1.

### 4.3. Spot-Plating Assay

Ten-fold diluted cultures were spotted onto LB plates supplemented with various amounts of arabinose to determine the level of heterologous expression of *luxCDABE* in *E. coli*, *P. aeruginosa*, and *P. putida*.

### 4.4. Luminescence Detection and Quantitation

After spotting the ten-fold diluted cultures, luminescence of the cells on plates was determined using the Tanon 5200 chemiluminescent imaging system (Tanon BioScience Pte Ltd., Shanghai, China). The exposure time was set at 10 s. When it exceeded the saturation point, the exposure time was reduced to 3 s. The luminescent image was quantified using ImageJ v1.48 software [41].

### 4.5. Extraction of Biosurfactants

Raw materials of RL and LOL were derived from the extraction of cell-free supernatants by using ethyl acetate after acidification and chloroform-methanol (2:1 *v*/*v*) solution, respectively. The raw materials were subsequently dissoled in methanol for TLC analysis or further purification using medium-pressure liquid chromatography (AKTA with a C8 column, Cytiva, Uppsala, Sweden). The AKTA-purified materials were utilized for CMC determination and LC-MS/MS analysis.

### 4.6. Thin-Layer Chromatography

Raw or purified RL and LOL were dissolved in methanol and spotted on TLC (type) plates that were subsequently developed with chloroform/methanol/acetic acid (70:10:1.4, *v*:*v*:*v*) and chloroform/methanol/water (70:25:4, *v*:*v*:*v*), respectively. RL and LOL on TLC were visualized using the anthrone–sulfuric acid and ninhydrin staining methods, respectively. For quantification of RL and LOL, quantitative standards of rhamnose (Cat.# 10030-85-0, Solarbio, Shanghai, China) and ornithine (Cat.# 3184-13-2, Macklin, Shanghai, China) were used to spot on TLC after development but prior to staining, respectively. Stained images were quantified using ImageJ v1.48 software [41]. The semi-quantitative method was validated using the AKTA-purified mRL or LOL.

### 4.7. Analysis of Surface Tension

Surface tension was determined by using the BZY-B surface tensiometer (Fangrui Instrument Co., Ltd., Shanghai, China) with the du Nouy ring method at 25 °C and calibrated using distilled water (72 mN/m) and ethanol (22 mN/m) prior to use.

### 4.8. Determination of Critical Micelle Concentration (CMC)

AKTA-purified mRL or LOL was dissolved in distilled water at the initial concentration of 10 mg L^−1^ and was subjected to two-fold serial dilution to a concentration of 10 μg L^−1^. Surface tension of the serial diluted solutions was determined by a surface tensiometer and plotted against solute concentration. CMC is a point at which reduced solute concentration cannot decrease the surface tension.

### 4.9. Analysis of Emulsification Capaci

The emulsification index E24% was analyzed according to a procedure reported previously [42]. In brief, equal amounts of two-fold diluted surfactant solutions (pH7) and crude oil were mixed using a vortex (IKA, Staufen, Germany) at the maximum level for 2 min and subsequently remained at a standstill for 24 h at 25 °C. The E24 index was estimated by a ratio between the emulsion volume and total content volume.

### 4.10. Liquid Chromatography Coupled with Tandem Mass Spectrometry (LC-MS/MS)

To analyze the composition of the rhamnolipids or lyso-ornithine lipid produced by KT2440, its AKTA-purified biosurfactant was resolved in chloroform at a concentration of 0.1 g/mL. LC–MS/MS analysis of the surfactants was performed using the Waters UPLC (Waters Corp., Milford, MA, USA) system equipped with the Aquity UPLC Beh-C18 column (1.7 μm, 2.1 × 50 mm; Waters Corp.) coupled with the AB Triple TOF 5600 plus System (AB Sciex, Framingham, MA, USA). In LC analysis, the mobile phases 0.1% formic acid–water (A) and 0.1% formic acid–acetonitrile (B) were employed. Linear gradient programs were set as follows, 0/20, 20/95,35/95, 36/20 (min/B%); sample injection volume was 2 μL; column oven temperature set as 35 °C; flow rate was 0.4 mL/min; and the UV detector was set at the wavelength of 220 nm. In MS analysis, the MS scan range was set at *m*/*z* 100–2000 in negative ion mode for rhamnolipid and positive mode for lyso-ornithine lipid with a source voltage of −4.5 kV and source temperature at 550 °C. The pressure of Gas 1 (Air) and Gas 2 (Air) were set to 50 psi. The pressure of Curtain Gas (N2) was set to 35 psi. Maximum allowed error was set to ± 5 ppm. Declustering potential (DP) was 100 V; collision energy (CE) was 10 V. For MS/MS acquisition mode, the parameters were almost the same except that the collision energy (CE) was set at 50 ± 20 V, ion release delay (IRD) at 67, and ion release width (IRW) at 25. Analyst TF 1.6 and Peakview v1.2 software (AB Sciex, Framingham, MA, USA) were used for data acquisition and data analysis, respectively.

## Data Availability

All data supporting the conclusion of this article are included within the manuscript and Supplementary Materials.

## Supplementary Materials

The following supporting information can be downloaded at: https://www.mdpi.com/article/10.3390/molecules29143288/s1, Figure S1: Real-time monitoring of luminescence reporter *luxCDABE* or *lux* controlled by P_BAD_ and P_BAD-SD_ in *E. coli* K12, Figure S2: Real-time monitoring of luminescence reporter *luxCDABE* or *lux* controlled by P_BAD_ and P_BAD-SD_ in *P. aeruginosa* PAO1, Figure S3: Real-time monitoring of luminescence reporter *luxCDABE* or *lux* controlled by P_BAD_ and P_BAD-SD_ in *P. putida* KT2440, Figure S4: mRL level in various AKTA fractions based on UV intensity and TLC analysis, Figure S5: pOEs-olsB plasmid but not ornithine is required for LOL production in KT2440, Figure S6: LOL level in various AKTA fractions based on UV intensity and TLC analysis.

## References

[1] Guzmán E., Ortega F., Rubio R.G. (2024). Exploring the world of rhamnolipids: A critical review of their production, interfacial properties, and potential application. Curr. Opin. Colloid Interface Sci..

[2] Marchant R., Banat I.M. (2012). Biosurfactants: A sustainable replacement for chemical surfactants?. Biotechnol. Lett..

[3] Biniarz P., Łukaszewicz M., Janek T. (2017). Screening concepts, characterization and structural analysis of microbial-derived bioactive lipopeptides: A review. Crit. Rev. Biotechnol..

[4] Fahy E., Cotter D., Sud M., Subramaniam S. (2011). Lipid classification, structures and tools. Biochim. Biophys. Acta.

[5] Santos D.K., Rufino R.D., Luna J.M., Santos V.A., Sarubbo L.A. (2016). Biosurfactants: Multifunctional biomolecules of the 21st century. Int. J. Mol. Sci..

[6] Maneerat S., Bamba T., Harada K., Kobayashi A., Yamada H., Kawai F. (2006). A novel crude oil emulsifier excreted in the culture supernatant of a marine bacterium, *Myroides* sp. strain SM1. Appl. Microbiol. Biotechnol..

[7] López-Lara I.M., Sohlenkamp C., Geiger O. (2003). Membrane lipids in plant-associated bacteria: Their biosyntheses and possible functions. Mol. Plant Microbe Interact..

[8] Knoche H.W., Shively J.M. (1972). The structure of an ornithine-containing lipid from *Thiobacillus thiooxidans*. J. Biol. Chem..

[9] Geiger O., Rohrs V., Weissenmayer B., Finan T.M., Thomas-Oates J.E. (1999). The regulator gene *phoB* mediates phosphate stress-controlled synthesis of the membrane lipid diacylglyceryl-N,N,N-trimethylhomoserine in *Rhizobium (Sinorhizobium) meliloti*. Mol. Microbiol..

[10] Gao J.-L., Weissenmayer B., Taylor A.M., Thomas-Oates J., López-Lara I.M., Geiger O. (2004). Identification of a gene required for the formation of lyso-ornithine lipid, an intermediate in the biosynthesis of ornithine-containing lipids. Mol. Microbiol..

[11] Weissenmayer B., Gao J.-L., López-Lara I.M., Geiger O. (2002). Identification of a gene required for the biosynthesis of ornithine-derived lipids. Mol. Microbiol..

[12] Williams W., Kunorozva L., Klaiber I., Henkel M., Pfannstiel J., Van Zyl L.J., Hausmann R., Burger A., Trindade M. (2019). Novel metagenome-derived ornithine lipids identified by functional screening for biosurfactants. Appl. Microbiol. Biotechnol..

[13] Kristoffersen V., Jenssen M., Jawad H.R., Isaksson J., Hansen E.H., Rämä T., Hansen K.Ø., Andersen J.H. (2021). Two Novel Lyso-Ornithine Lipids Isolated from an Arctic Marine *Lacinutrix* sp. Bacterium. Molecules.

[14] Guzman L.-M., Belin D., Carson M.J., Beckwith J. (1995). Tight Regulation, Modulation, and High-Level Expression by Vectors Containing the Arabinose PBAD Promoter. J. Bacteriol..

[15] Lane M.C., Alteri C.J., Smith S.N., Mobley H.L. (2007). Expression of flagella is coincident with uropathogenic *Escherichia coli* ascension to the upper urinary tract. Proc. Natl. Acad. Sci. USA.

[16] Kovach M.E., Elzer P.H., Hill D.S., Robertson G.T., Farris M.A., Roop R.M., Peterson K.M. (1995). Four new derivatives of the broad-host-range cloning vector pBBR1MCS, carrying different antibiotic-resistance cassettes. Gene.

[17] Nishikubo T., Nakagawa N., Kuramitsu S., Masui R. (2005). Improved heterologous gene expression in *Escherichia coli* by optimizition of the AT-content of codons immediately downstream of the initiation codon. J. Biotechnol..

[18] Soberon-Chavez G., Gonzalez-Valdez A., Soto-Aceves M.P., Cocotl-Yanez M. (2021). Rhamnolipids produced by *Pseudomonas*: From molecular genetics to the market. Microb. Biotechnol..

[19] Cho C.H., Lee S.B. (2018). Comparison of clinical characteristics and antibiotic susceptibility between *Pseudomonas aeruginosa* and *P. putida* keratitis at a tertiary referral center: A retrospective study. BMC Ophthalmol..

[20] Cheng T., Liang J., He J., Hu X., Ge Z., Liu J. (2017). A novel rhamnolipid-producing *Pseudomonas aeruginosa* ZS1 isolate derived from petroleum sludge suitable for bioremediation. AMB Expr..

[21] Wittgens A., Santiago-Schuebel B., Henkel M., Tiso T., Blank L.M., Hausmann R., Hofmann D., Wilhelm S., Jaeger K.E., Rosenau F. (2018). Heterologous production of long-chain rhamnolipids from *Burkholderia glumae* in *Pseudomonas putida*—A step forward to tailor-made rhamnolipids. Appl. Microbiol. Biotechnol..

[22] Dulcey C.E., de los Santos Y.L., Létourneau M., Déziel E., Doucet N. (2019). Semi-rational evolution of the 3-(3-hydroxyalkanoyloxy)alkanoate (HAA) synthase RhlA to improve rhamnolipid production in *Pseudomonas aeruginosa* and *Burkholderia glumae*. FEBS J..

[23] Germer A., Tiso T., Müller C., Behrens B., Vosse C., Scholz K., Froning M., Hayen H., Blank L.M. (2020). Exploiting the Natural Diversity of RhlA Acyltransferases for the Synthesis of the Rhamnolipid Precursor 3-(3-Hydroxyalkanoyloxy)Alkanoic Acid. Appl. Environ. Microbiol..

[24] Vences-Guzman M.A., Geiger O., Sohlenkamp C. (2012). Ornithine lipids and their structural modifications: From A to E and beyond. FEMS Microbiol. Lett..

[25] Winsor G.L., Griffiths E.J., Lo R., Dhillon B.K., Shay J.A., Brinkman F.S. (2006). Enhanced annotations and features for comparing thousands of *Pseudomonas* genomes in the *Pseudomonas* genome database. Nucleic Acids Res..

[26] Tian L., Yang Z., Wang J., Liu J. (2023). Analysis of the plasmid-based ts-mutant Δ*fabA/pTS-fabA* reveals its lethality under aerobic growth conditions that is suppressed by mild overexpression of *desA* at a restrictive temperature in *Pseudomonas aeruginosa*. Microbiol. Spectr..

[27] Tiso T., Sabelhaus P., Behrens B., Wittgens A., Rosenau F., Hayen H., Blank L.M. (2016). Creating metabolic demand as an engineering strategy in *Pseudomonas putida*—Rhamnolipid synthesis as an example. Metab. Eng. Commun..

[28] Siegele D.A., Hu J.C. (1997). Gene expression from plasmids containing the araBAD promoter at subsaturating inducer concentrations represents mixed populations. Proc. Natl. Acad. Sci. USA.

[29] Shilling P.J., Khananisho D., Cumming A.J., Söderström B., Daley D.O. (2022). Signal amplification of araC pBAD using a standardized translation initiation region. Synth. Biol..

[30] Setoodeh P., Jahanmiri A., Eslamloueyan R., Niazi A., Ayatollahi S.S., Aram F., Mahmoodi M., Hortamani A. (2014). Statistical screening of medium components for recombinant production of *Pseudomonas aeruginosa* ATCC 9027 rhamnolipids by nonpathogenic cell factory *Pseudomonas putida* KT2440. Mol. Biotechnol..

[31] Wittgens A., Kovacic F., Müller M.M., Gerlitzki M., Santiago-Schübel B., Hofmann D., Tiso T., Blank L.M., Henkel M., Hausmann R. (2017). Novel insights into biosynthesis and uptake of rhamnolipids and their precursors. Appl. Microbiol. Biotechnol..

[32] Tiso T., Ihling N., Kubicki S., Biselli A., Schonhoff A., Bator I., Thies S., Karmainski T., Kruth S., Willenbrink A.L. (2020). Integration of Genetic and Process Engineering for Optimized Rhamnolipid Production Using *Pseudomonas putida*. Front. Bioeng. Biotechnol..

[33] Weihmann R., Kubicki S., Bitzenhofer N.L., Domröse A., Bator I., Kirschen L.M., Kofler F., Funk A., Tiso T., Blank L.M. (2022). The modular pYT vector series employed for chromosomal gene integration and expression to produce carbazoles and glycolipids in *P. putida*. FEMS Microbes..

[34] Brandy S.F., Clardy J. (2000). Long-chain *N*-acyl amino acid antibiotics isolated from heterologously expressed environmental DNA. J. Am. Chem. Soc..

[35] Cho W., York A.G., Wang R., Wyche T.P., Piizzi G., Flavell R.A., Crawford J.M. (2022). N-Acyl Amides from *Neisseria meningitidis* and Their Role in Sphingosine Receptor Signaling. Chembiochem.

[36] Haeger G., Jolmes T., Oyen S., Jaeger K.E., Bongaerts J., Schörken U., Siegert P. (2024). Novel recombinant aminoacylase from *Paraburkholderia monticola* capable of N-acyl-amino acid synthesis. Appl. Microbiol. Biotechnol..

[37] Urum K., Pekdemir T. (2004). Evaluation of biosurfactants for crude oil contaminated soil washing. Chemosphere.

[38] Ramirez D., Shaw L.J., Collins C.D. (2021). Oil sludge washing with surfactants and co-solvents: Oil recovery from different types of oil sludges. Environ. Sci. Pollut. Res..

[39] Bertani G. (2004). Lysogeny at mid-twentieth century: P1, P2, and other experimental systems. J. Bacteriol..

[40] Zajic E., Supplison B. (1972). Emulsification and degradation of “Bunker C” fuel oil by microorganisms. Biotechnol. Bioeng..

[41] Schneider C.A., Rasband W.S., Eliceiri K.W. (2012). NIH Image to ImageJ: 25 years of image analysis. Nat. Methods.

[42] Cooper D.G., Goldenberg B.G. (1987). Surface-active agents from two Bacillus species. Appl. Environ. Microbiol..

