# Supplementary material for: Optimizing Systems for Robust Heterologous Production of Biosurfactants Rhamnolipid and Lyso-Ornithine Lipid in Pseudomonas putida KT2440
Source: Molecules. 2024 Jul 11;29(14):3288. doi: 10.3390/molecules29143288 (PMC11279095; doi:10.3390/molecules29143288)

Figure S1. Real-time monitoring of luminescence reporter *luxCDABE* or *lux* controlled by P_{BAD} and $P_{BAD\text{-}SD}$ in *E. coli* K12. (A) A representative images of cell growth at 3h, 6h, 12h, and 24h after spotted on to plate. Ten-fold serial diluted cultures are spotted on plates supplemented with or without arabinose. pOE, pOE-lux, and pOEs-lux stand for *E. coli* K12/pOE (blank vector), K12/pBAD-lux, and K12/pBAD-SD-lux strains. (B) Luminescence detected from plates supplemented with or without arabinose at various time points as indicated at right side. Exposure time for the luminescence is 10s.

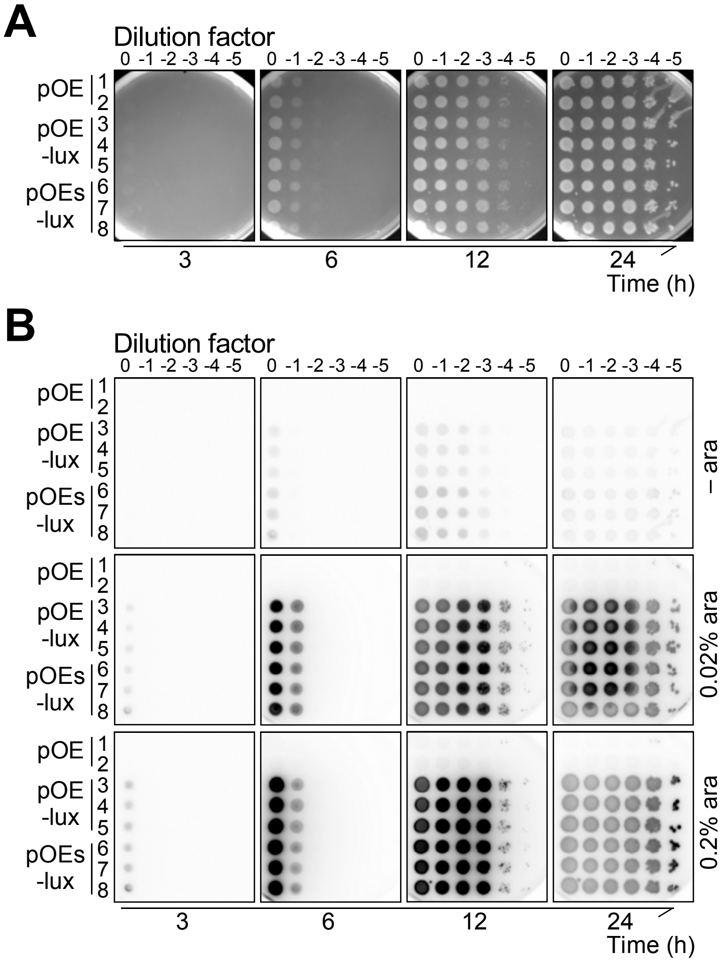

Supplement: Supplementary file 1 [file molecules-29-03288-s001.zip › Figure S1.pdf]
