# Supplementary material for: Optimizing Systems for Robust Heterologous Production of Biosurfactants Rhamnolipid and Lyso-Ornithine Lipid in Pseudomonas putida KT2440
Source: Molecules. 2024 Jul 11;29(14):3288. doi: 10.3390/molecules29143288 (PMC11279095; doi:10.3390/molecules29143288)

Figure S3. Real-time monitoring of luminescence reporter *luxCDABE* or *lux* controlled by PBAD and PBAD-SD in *P. putida* KT2440. The display in (A) and (B) is identical to Figure S1. (C) Luminescence detected from plates supplemented with 0.2% arabinose. Exposure time for luminescence is decreased to 3s instead of 10s as in (B).

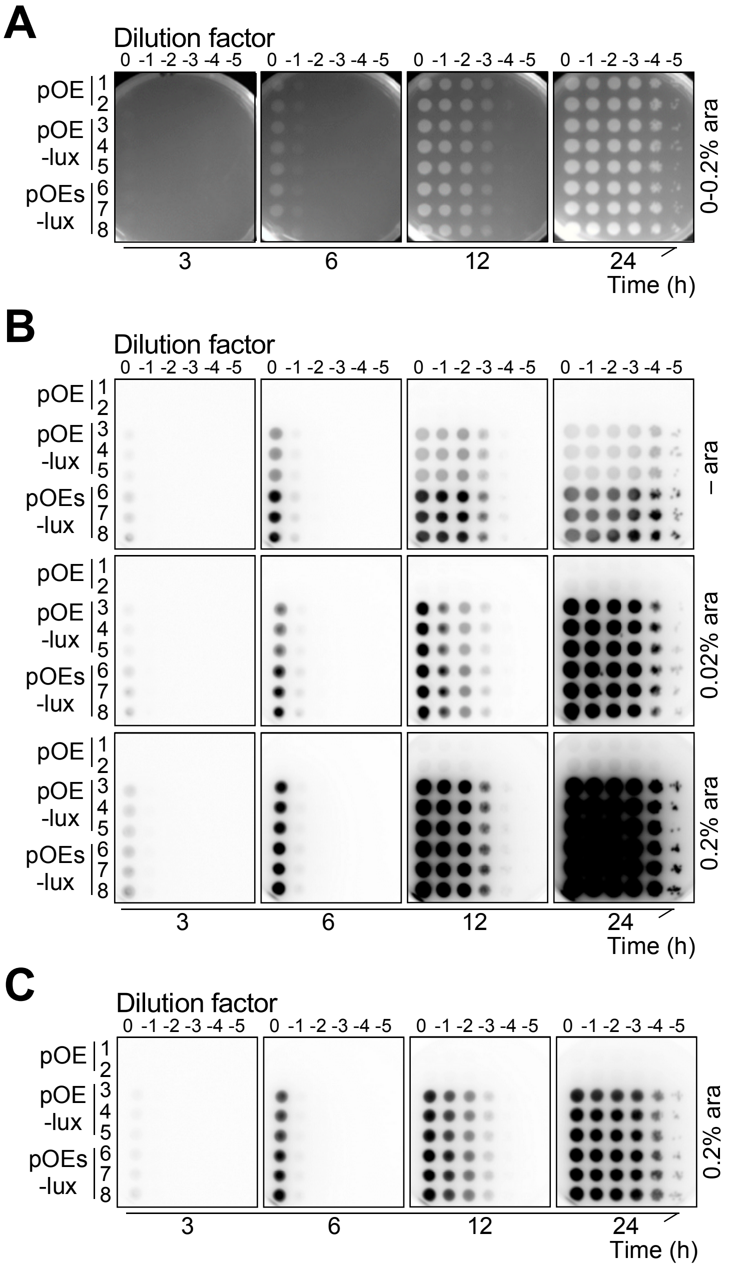

Supplement: Supplementary file 1 [file molecules-29-03288-s001.zip › Figure S3.pdf]
