# Supplementary material for: Optimizing Systems for Robust Heterologous Production of Biosurfactants Rhamnolipid and Lyso-Ornithine Lipid in Pseudomonas putida KT2440
Source: Molecules. 2024 Jul 11;29(14):3288. doi: 10.3390/molecules29143288 (PMC11279095; doi:10.3390/molecules29143288)
Supplement: Supplementary file 1 [file molecules-29-03288-s001.zip › Figure S4.pdf]

Figure S4. mRL level in various AKTA fractions based on UV intensity and TLC analysis (see Figure 4A) is corelated with their oil-spreading activities. Oil-spreading images with various fractions indicated are shown.

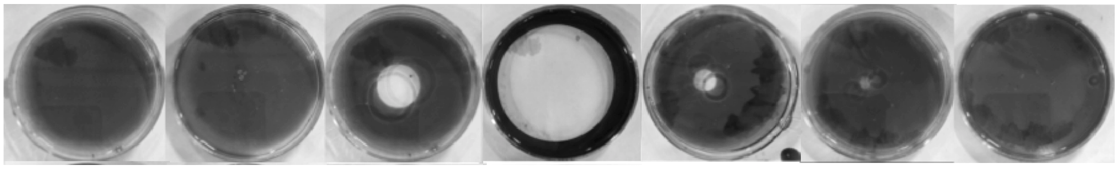

7 8 9 10 11 12 13
