# Supplementary material for: Optimizing Systems for Robust Heterologous Production of Biosurfactants Rhamnolipid and Lyso-Ornithine Lipid in Pseudomonas putida KT2440
Source: Molecules. 2024 Jul 11;29(14):3288. doi: 10.3390/molecules29143288 (PMC11279095; doi:10.3390/molecules29143288)

Figure S5. pOEs-olsB plasmid but not ornithine is required for LOL production in KT2440. (A) LOL is detected in culture of KT2440/pOEs-olsB. (B) LOL is hradly detected in culture of KT2440/pOEs.

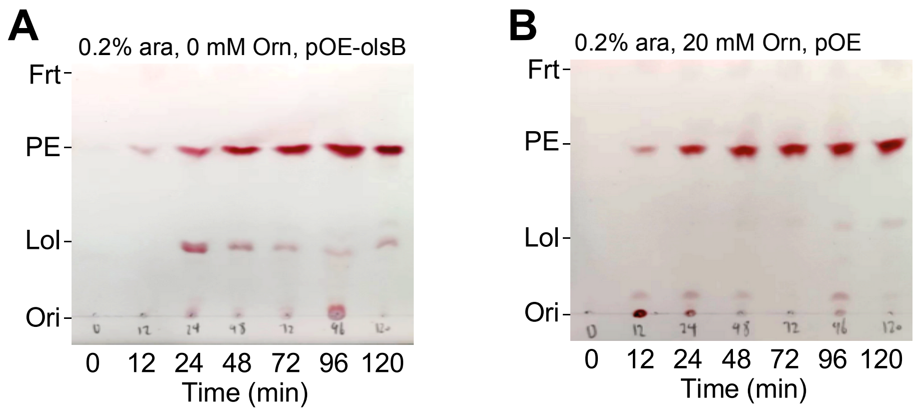

Supplement: Supplementary file 1 [file molecules-29-03288-s001.zip › Figure S5.pdf]
